# A Fibrinogen‐Mimicking, Activated‐Platelet‐Sensitive Nanocoacervate Enhances Thrombus Targeting and Penetration of Tissue Plasminogen Activator for Effective Thrombolytic Therapy

**DOI:** 10.1002/adhm.202201265

**Published:** 2022-07-31

**Authors:** Yu Huang, Jingxuan Jiang, Jie Ren, Yuanyuan Guo, Qianqian Zhao, Jia Zhou, Yuehua Li, Rongjun Chen

**Affiliations:** ^1^ Department of Radiology Shanghai Jiao Tong University Affiliated Sixth People's Hospital 600 Yi Shan Road Shanghai 200233 P. R. China; ^2^ Department of Chemical Engineering Imperial College London South Kensington Campus London SW7 2AZ UK

**Keywords:** chitosan, fibrinogen‐mimicking nanocoacervate, targeted thrombolysis, thrombus penetration, tissue plasminogen activators

## Abstract

The development of a fibrinolytic system with long circulation time, high thrombus targeting, efficient thrombus penetration, effective thrombolysis, and minimal hemorrhagic risk remains a major challenge. Herein, inspired by fibrinogen binding to activated platelets in thrombosis, this article reports a fibrinogen‐mimicking, activated‐platelet‐sensitive nanocoacervate to enhance thrombus penetration of tissue plasminogen activator (tPA) for targeted thrombolytic therapy. This biomimetic nanothrombolytic system, denoted as RGD‐Chi@tPA, is constructed by “one‐pot” coacervation through electrostatic interactions between positively charged arginine‐glycine‐aspartic acid (RGD)‐grafted chitosan (RGD‐Chi) and negatively charged tPA. Flow cytometry and confocal laser scanning microscopy measurements show targeting of RGD‐Chi@tPA to activated platelets. Controlled tPA release triggered by activated platelets at a thrombus site is demonstrated. Its targeted fibrinolytic and thrombolytic activities are measured in in vitro models. The pharmacokinetic profiles show that RGD‐Chi@tPA can significantly prolong circulation time compared to free tPA. In a mouse tail thrombus model, RGD‐Chi@tPA displays efficient thrombus targeting and penetration, enabling a complete vascular recanalization as confirmed by the fluorescence imaging, histochemical assay, and laser speckle contrast imager. Consequently, RGD‐Chi@tPA induces a substantial enhancement in thrombolysis with minimal hemorrhagic risk compared to free tPA. This simple, effective, and safe platform holds great promise for the development of thrombolytic nanomedicines.

## Graphical abstract

Inspired by fibrinogen binding to activated platelets in thrombosis, a fibrinogen‐mimicking, activated‐platelet‐sensitive nanocoacervate (RGD‐Chi@tPA) is developed by “one‐pot” coacervation through simple electrostatic interaction between positively charged arginine‐glycine‐aspartic acid‐grafted chitosan and negatively charged tissue plasminogen activator to enhance thrombus targeting and penetration for effective thrombolytic therapy.

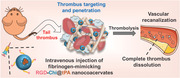

## 1 Introduction

Thrombosis is the generation of a thrombus within a vessel and is naturally regulated by homeostasis between coagulation and fibrinolysis.^[^ ^1^ ^]^ Breakage of this balance can cause pathological thromboembolism, leading to fatal arteriovenous syndromes such as ischemic stroke, acute myocardial infarction, and pulmonary embolism.^[^ ^2^, ^3^, ^4^ ^]^ The mainstay of treatment of these thrombotic disorders is rapid recanalization of blood vessels by angioplasty and thrombectomy.^[^ ^5^ ^]^ Since the angioplastic and surgical interventions are costly and depend on the accessibility of specialized healthcare equipment and patients’ health conditions, a pharmacologic intervention with intravenous infusion of fibrinolytic enzymes, such as tissue plasminogen activator (tPA), streptokinase (SK), and urokinase (UK), is a widely accepted medical practice for treating thrombi.^[^ ^6^, ^7^, ^8^ ^]^ Currently available fibrinolytic enzymes act by catalyzing plasminogen into plasmin, which in turn, dissolves a thrombus via fibrinolysis.^[^ ^9^ ^]^ However, due to the short circulation half‐life and limited thrombus specificity, a frequent high‐dose systemic administration of fibrinolytic enzymes is compelled to be used to achieve efficient thrombolysis, resulting in indiscriminate catalytic activity on circulating plasminogen and subsequent generation of off‐target plasmin that can cause systemic hemorrhage.^[^ ^10^, ^11^, ^12^ ^]^ Meanwhile, poor thrombus penetration of native fibrinolytic enzymes is another major challenge. This is because the thrombus front comprising of the fibrin network and plasma proteins can limit the transport of fibrinolytic enzymes to the thrombus interior and confine thrombolytic activity on the thrombus surface, consequently diminishing thrombolytic efficacy.^[^ ^13^, ^14^, ^15^, ^16^ ^]^

To date, a variety of fibrinolytic enzyme delivery systems have attempted to solve the above challenges.^[^ ^17^, ^18^, ^19^, ^20^, ^21^, ^22^, ^23^ ^]^ For example, Jin et al.^[^ ^24^ ^]^ and Berger et al.^[^ ^25^ ^]^ reported a PEGylated delivery system by direct conjugation of lumbrokinase (LK) and tPA enzymes, respectively, with polyethylene glycol (PEG) to prolong the circulation half‐life. Absar et al. demonstrated a camouflaged tPA delivery system by conjugating tPA with human serum albumin followed by surface decoration with a thrombus‐homing peptide (CQQHHLGGAKQAGDV) for thrombus‐targeted delivery of tPA.^[^ ^26^ ^]^ Chung et al.^[^ ^27^ ^]^ and Wang et al.^[^ ^28^ ^]^ reported tPA‐encapsulated poly‐(lactide‐coglycolide) nanoparticles with a surface coating of chitosan for accelerated thrombolysis through enhanced thrombus penetration due to the synergistic effects of the nano size of the system and electrostatic adherence between positively charged chitosan on the nanoparticle surface and negatively charged components in a thrombus. While those efforts were encouraging, they only partially addressed the aforementioned challenges and suffered from a reduction of fibrinolytic activity resulting from direct conjugation.^[^ ^29^, ^30^, ^31^ ^]^ Despite the nanoparticulate systems coated with positively charged chitosan being demonstrated to improve penetration of fibrinolytic enzymes into a thrombus, a triggered release of the drug, specifically at a thrombus site, remains a major challenge.^[^ ^32^ ^]^ Most recently, inspired by the “bridging effect” between fibrinogen and activated platelets at a thrombus site, we reported fibrinogen‐mimicking, multiarm lipid nanovesicles coated with PEG that was terminally conjugated with a cyclic arginine‐glycine‐aspartic acid (cRGD) peptide, for highly efficient thrombolysis through selective binding to activated platelets and triggered tPA release at a thrombus site under static and physiological flow conditions.^[^ ^33^, ^34^ ^]^ Although this approach is considered to be a notable advancement toward localized fibrinolytic action, its thrombus penetration and thrombolytic processes have not been tested in an animal thrombus model yet. Therefore, it is attractive to develop an “all‐in‐one” fibrinolytic system that presents a long circulation half‐life, high thrombus selectivity, triggered drug release, and efficient thrombus penetration, thus enabling effective thrombolytic therapy with minimal risk of hemorrhage.

**Scheme 1:**
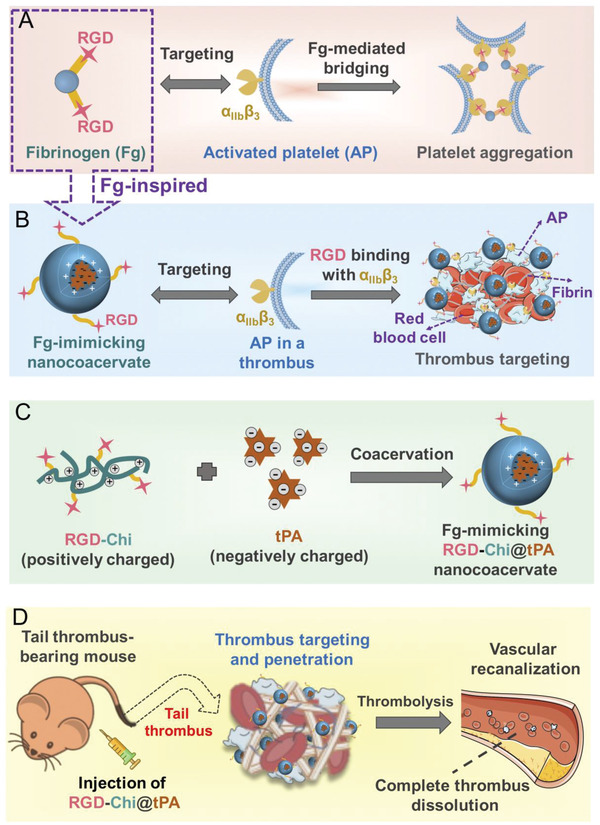
A) Schematic illustration of targeting of RGD ligands to fibrinogen (Fg) and *α*_IIb_ *β*_3_ integrin on activated platelets (APs). B) Schematic illustration of thrombus targeting of a fibrinogen‐mimicking nanocoacervate through selective binding of RGD ligands to *α*_IIb_ *β*_3_ integrin on activated platelets at a thrombus site. C) Schematic illustration of the preparation of fibrinogen‐mimicking nanocoacervates, RGD‐Chi@tPA, by coacervation through electrostatic interactions between positively charged RGD‐grafted chitosan (RGD‐Chi) and negatively charged tPA. D) Schematic illustration of targeted thrombolysis after intravenous injection of RGD‐Chi@tPA in a mouse tail thrombus model.

Herein, we rationally designed a fibrinogen‐mimicking, activated‐platelet‐sensitive nanocoacervate, denoted as RGD‐Chi@tPA, to achieve enhanced thrombus penetration of tPA for targeted thrombolytic therapy (**Scheme** **1**A,B). tPA was selected as a model thrombolytic drug, considering it is the only U.S. Food and Drug Administration‐approved thrombolytic agent for stroke but limited by its short circulation time (only 2 to 6 min) and significant off‐target hemorrhagic side effects.^[^ ^6^ ^]^ This “all‐in‐one” thrombolytic system was prepared by a robust “one‐pot” coacervation process through electrostatic interactions between positively charged RGD‐grafted chitosan (RGD‐Chi) and negatively charged tPA (Scheme 1C).^[^ ^35^, ^36^, ^37^ ^]^ The enzymatic activity of tPA could be retained due to the involvement of tPA in the self‐assembly without a need for conjugation and entrapment in the nanocoacervates would protect tPA from premature inactivation or removal while in the blood circulation, thus allowing the prolonged retention time in blood and minimal undesirable bleeding side effects.^[^ ^38^ ^]^ The thrombus targeting was rendered by side chain modification of chitosan (an amino polysaccharide) with fibrinogen‐derived RGD ligands that could specifically bind to stimulated GPIIb/IIIa (*α*_IIb_ *β*_3_) integrins abundantly expressed on the surface of activated platelets at a thrombus site (Scheme 1A).^[^ ^39^, ^40^ ^]^ Activated platelets are ideal receptors for targeting thrombi since aggregation of activated platelets mediated by fibrinogens is a significant hallmark event in thrombosis (Scheme 1A,B).^[^ ^41^, ^42^, ^43^ ^]^ To facilitate thrombus penetration, the nanocoacervate size was carefully controlled since nano‐sized particles (250–320 nm) were reported to penetrate the porous structure of fibrin gels and blood clots.^[^ ^44^, ^45^ ^]^ The positive charges resulting from chitosan in the designed nanocoacervates would further deepen thrombus penetration by electrostatic adsorption to the negatively charged compositions inside a thrombus.^[^ ^27^, ^28^ ^]^ In addition, the thrombus targeting of nanocoacervates would further enhance the thrombus penetration through interactions between RGD ligands on the nanocoacervate surface and *α*_IIb_ *β*_3_ integrins on the activated platelet surface inside a thrombus. Building on these design principles, our central hypothesis was that the intravenously administered fibrinogen‐mimicking nanocoacervate, RGD‐Chi@tPA, in an animal thrombus model could selectively anchor onto not only the surface but the interior of the thrombus, where tPA release could be triggered locally, leading to the enhanced targeted thrombolysis and efficient vascular recanalization with minimal unwanted side effects (Scheme 1D).

## 2 Results and Discussion

### 2.1 Preparation and Characterization of RGD‐Chi@tPA

**Figure 1:**
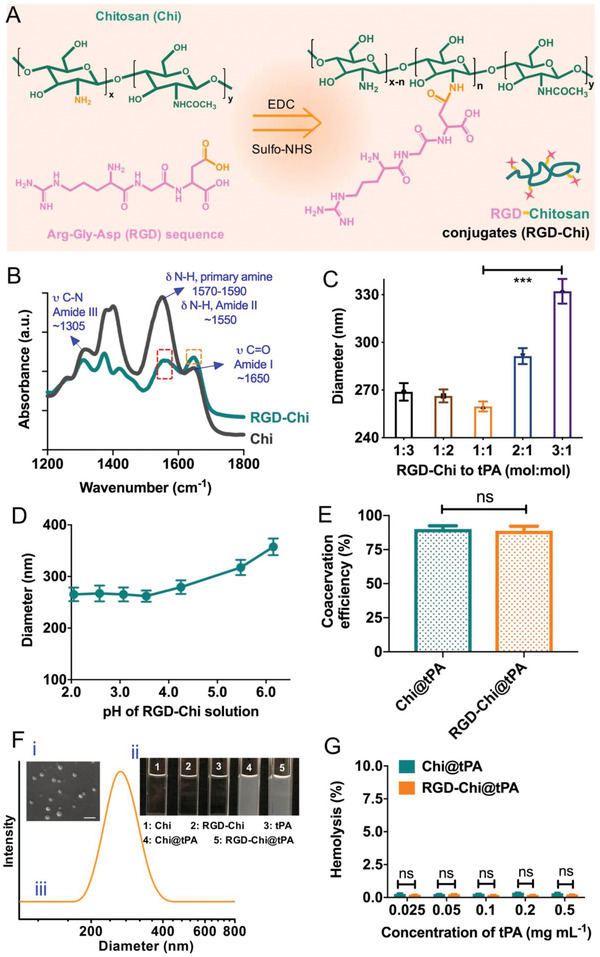
A) Synthesis of the RGD‐Chi conjugate from chitosan (Chi) and the RGD peptide. B) FTIR spectra of Chi and RGD‐Chi. C) Mean hydrodynamic sizes of RGD‐Chi@tPA as a function of the molar ratio of RGD‐Chi to tPA (RGD‐Chi solution at pH 3.5 and 25 °C). D) Mean hydrodynamic sizes of RGD‐Chi@tPA as a function of pH of the RGD‐Chi solution (1:1 molar ratio of RGD‐Chi to tPA and 25 °C). E) Coacervation efficiencies of Chi@tPA and RGD‐Chi@tPA. F) i) SEM micrograph of RGD‐Chi@tPA (scale bar: 500 nm); ii) Photographs of Chi, RGD‐Chi, tPA, Chi@tPA, and RGD‐Chi@tPA solutions; iii) Typical intensity‐weighted DLS plot of RGD‐Chi@tPA in aqueous solution (pH 7.4 and 25 °C). G) Hemolysis of Chi@tPA and RGD‐Chi@tPA, respectively, as a function of tPA concentration. Data are presented as the average ± standard deviation (*n* = 3). Statistical analysis was performed by the Student's *t*‐test (***p < 0.001 and ns, not significant).

The RGD‐Chi conjugate (**Figure** **1**A) was first synthesized through covalent conjugation of the carboxylic group on the RGD peptide to the amine group on chitosan through the EDC/NHS coupling chemistry.^[^ ^46^ ^]^ The Fourier transform infrared (FTIR) spectra presented in Figure 1B shows the typical peaks associated with the amide formation.^[^ ^47^ ^]^ For Chi, the absorption peaks at about 1650, 1550, and 1305 cm^−1^ were assigned to C═O stretching vibrations of the amide I band, N—H bending vibrations of the amide II band, and C—N stretching vibrations of the amide III band, respectively. N—H bending vibrations of the deacetylated primary amine (—NH_2_, 1590–1570 cm^−1^) and the amide II band were overlapped.^[^ ^47^ ^]^ After the EDC‐NHS mediated coupling reaction, the vibrational band that corresponds to primary amine groups (red box) diminished, while the absorption band at about 1650 cm^−1^ (orange box, C═O stretching vibrations of the amide I band) increased, confirming the successful formation of new amide bonds.^[^ ^46^ ^]^ The RGD‐Chi product was further confirmed by proton nuclear magnetic resonance spectroscopy (^1^H NMR) (Figures S1–S3, Supporting Information). The weight percentage of RGD was 15.6 ± 1.3 wt%, as calculated by the bicinchoninic acid (BCA) assay.^[^ ^48^ ^]^

As shown in Figure S4, Supporting Information, RGD‐Chi had a positive charge due to the protonation of the remaining amino groups on RGD‐Chi.^[^ ^27^, ^28^ ^]^ Mixing of anionic tPA with cationic RGD‐Chi resulted in the formation of RGD‐Chi@tPA nanocoacervates with a lower positive surface charge. Figure S5, Supporting Information, shows a slight red‐shift of the peak (≈280 nm) and peak broadening in the UV–vis spectrum of RGD‐Chi@tPA as compared to that of tPA, further confirming the presence of electrostatic interactions between RGD‐Chi and tPA upon mixing and the subsequent formation of RGD‐Chi@tPA nanocoacervates.^[^ ^49^ ^]^

Coacervation parameters were investigated to control the particle size of the nanocoacervates. As shown in Figure 1C, with increasing the molar ratio of RGD‐Chi to tPA from 1:3 to 1:1, there was a marginal decrease in hydrodynamic size from 266.9 ± 5.5 to 259.7 ± 3.1. A further increase in the molar ratio to 3:1 led to a significantly increased particle size to 332.1 ± 7.7. This suggests that 1:1 was the optimal ratio where all cationic RGD‐Chi fully interacted with anionic tPA whilst the presence of excess RGD‐Chi resulted in larger‐sized nanocoacervates at higher ratios. Similarly, the polydispersity index (PDI) of the nanocoacervates was the lowest when the molar ratio of RGD‐Chi to tPA was 1:1 (Figure S6, Supporting Information). The negative logarithm of the acid dissociation constant (pKa) value of the amine group in chitosan is ≈6.3.^[^ ^50^ ^]^ With decreasing the pH of RGD‐Chi solution from 6.15 to 3.54, the degree of protonation of amino groups on RGD‐Chi was increased, which resulted in a decrease in mean nanocoacervate size from 357.4 ± 16.1 to 261.9 ± 10.9 (Figure 1D) due to stronger electrostatic interactions between cationic RGD‐Chi and anionic tPA.^[^ ^51^, ^52^ ^]^ No significant size change was observed when the pH was further decreased from 3.54 to 2.05. Figure S7, Supporting Information, shows the lowest PDI for nanocoacervates prepared with the RGD‐Chi solution at the optimal pH of 3.54 within the same pH range tested. As shown in Figure 1E and Figure S8, Supporting Information, the coacervation efficiency (CE) and tPA encapsulation efficiency (EE) of RGD‐Chi@tPA prepared under the optimal conditions were 88.7 ± 3.4% and 53.6 ± 3.3%, respectively, comparable to those of Chi@tPA without RGD coating (90.1 ± 2.3% and 51.8 ± 5.4%, respectively).

SEM micrographs in Figure 1Fi and Figure S9, Supporting Information, confirmed that the Chi@tPA and RGD‐Chi@tPA nanocoacervates were spherical in shape with a similar diameter. As shown in Figure 1Fii, the respective clear solutions of Chi, RGD‐Chi, and tPA were observed, whereas an opalescent suspension was formed spontaneously when tPA was mixed with the Chi or RGD‐Chi solution, respectively, indicative of successful coacervation through electrostatic interactions between cationic Chi or RGD‐Chi and anionic tPA.^[^ ^53^ ^]^ DLS measurements, shown in Figure 1Fiii and Figure S10, Supporting Information, further confirmed that the hydrodynamic size of RGD‐Chi@tPA (259.3 ± 5.2 nm) was similar to that of Chi@tPA (262.7 ± 4.5 nm). This suggests that RGD conjugation onto chitosan caused negligible changes in particle size of nanocoacervates. A hemolysis model was used to investigate the hemocompatibility of the nanocoacervates. As shown in Figure 1G and Figure S11, Supporting Information, the level of hemolysis was less than 0.3% within the concentration ranges of Chi and RGD‐Chi (up to 1 mg mL^−1^), and the Chi@tPA and RGD‐Chi@tPA nanocoacervates (up to an equivalent tPA concentration of 0.5 mg mL^−1^) tested, suggesting that the two starting materials and two nanocoacervates had high biocompatibility in blood.

The platelet aggregation assay was carried out to examine the possibility of nonspecific platelet activation by RGD‐Chi@tPA. As shown in Figure S12A, Supporting Information, in the absence of thrombin as an agonist, the purified platelet suspension with or without the RGD‐Chi@tPA (tPA dose of 0.2 mg mL^−1^) pre‐incubation displayed negligible aggregation at ≈7.9% (black curve) and ≈10.8% (yellow curve), respectively, after 15 min. By comparison, in the presence of thrombin (1 U mL^−1^), the non‐distinguishable high levels of platelet aggregation at ≈93.4% (red curve) and ≈94.7% (blue curve) were observed after 15 min for the purified platelet suspension with or without the RGD‐Chi@tPA (tPA dose of 0.2 mg mL^−1^) pre‐incubation, respectively. Furthermore, the platelet aggregation behavior in the absence of an agonist was investigated with increasing amounts of RGD‐Chi@tPA. Figure S12B, Supporting Information, shows no significant increase in the percentage of maximum platelet aggregation with increasing the tPA dose to 0.5 mg mL^−1^. Those results suggest that the RGD‐Chi@tPA nanocoacervate did not induce obvious nonspecific activation of platelets and did not alter the ability of platelets to aggregate as well upon simulation by an agonist.

### 2.2 Selective Binding to Activated Platelets and Triggered tPA Release at a Thrombus Site

**Figure 2:**
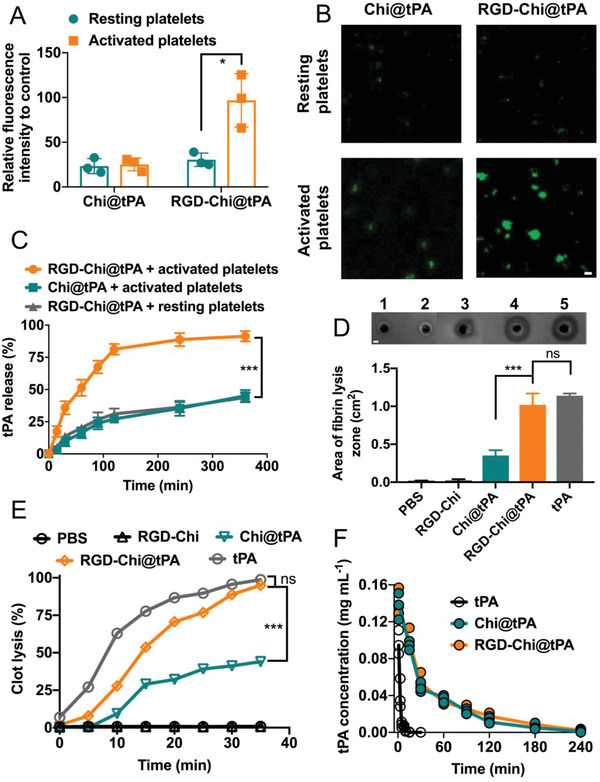
A) Relative mean fluorescence intensities of resting and activated platelets treated with the FITC‐labeled Chi@tPA and RGD‐Chi@tPA, respectively, as measured by flow cytometry. B) CLSM images of resting and activated platelets incubated with the FITC‐labeled Chi@tPA and RGD‐Chi@tPA, respectively (scale bar: 5 µm). C) tPA release profiles of Chi@tPA and RGD‐Chi@tPA after incubation with resting or activated platelets. D) Calculated areas of fibrin clot lysis zone after treatment with PBS buffer, RGD‐Chi, Chi@tPA, RGD‐Chi@tPA, and free tPA, respectively, after treatment with activated platelets for 2 h. Inset: representative photograph of fibrin lysis zone after treatment with the No. ≈1–5 formulations: PBS buffer, RGD‐Chi, Chi@tPA, RGD‐Chi@tPA, and free tPA, respectively (scale bar: 4 mm). E) Time‐dependent blood clot lysis in the halo model after treatment with PBS buffer, RGD‐Chi, Chi@tPA, RGD‐Chi@tPA, and free tPA, respectively. F) Pharmacokinetic profiles of Chi@tPA, RGD‐Chi@tPA, and free tPA in healthy SD rats. Data are presented as the average ± standard deviation (*n* = 3). Statistical analysis was performed by the Student's *t*‐test and ANOVA test (*p < 0.05, ***p < 0.001, and ns, not significant).

Figure S13, Supporting Information, compares the binding affinities of the fluorescein isothiocyanate (FITC)‐labeled Chi@tPA and RGD‐Chi@tPA to resting and activated platelets, respectively, by flow cytometry. As shown in Figure S13A, Supporting Information, resting platelets treated with the FITC‐labeled Chi@tPA and RGD‐Chi@tPA displayed a similar level of fluorescence intensity, which was only a small increase compared to the control (resting platelets treated with pH 7.4 phosphate‐buffered saline [PBS] buffer alone). This suggests that the Chi@tPA and RGD‐Chi@tPA nanocoacervates have a low level of attachment to resting platelets. Figure S13B, Supporting Information, shows that activated platelets treated with the FITC‐labeled Chi@tPA displayed only a small increase in fluorescence intensity. As a comparison, the FITC‐labeled RGD‐Chi@tPA bound more avidly to activated platelets. **Figure** **2**A shows that the fluorescence intensity of activated platelets treated with the FITC‐labeled RGD‐Chi@tPA was about threefold higher than that treated with the FITC‐labeled Chi@tPA. These results suggest that RGD peptides can efficiently facilitate the targeting of RGD‐Chi@tPA to activated platelets. Furthermore, the selective binding of the FITC‐labelled RGD‐Chi@tPA to activated platelets was validated by confocal laser scanning microscopy (CLSM). As shown in Figure 2B, resting platelets treated with the FITC‐labeled Chi@tPA and RGD‐Chi@tPA, respectively, showed negligible green fluorescence. However, significantly enhanced staining of activated platelets by the FITC‐labeled RGD‐Chi@tPA was observed as compared to those treated with the FITC‐labeled Chi@tPA without RGD peptides. Quantitative analysis of the mean fluorescence intensity in those images (Figure S14, Supporting Information) suggests that RGD‐Chi@tPA showed an approximately threefold enhancement in binding affinity to activated platelets as compared to Chi@tPA. This is in good agreement with the flow cytometry results shown in Figure 2A, further consolidating that RGD‐Chi@tPA can efficiently bind to activated platelets at a thrombus site.^[^ ^33^, ^34^ ^]^

Triggered release of tPA specifically at a thrombus site is critical for targeted thrombolysis. As shown in Figure 2C, upon binding of RGD‐Chi@tPA to activated platelets, around 50% of the entrapped tPA was released within 1 h and the tPA release continued to increase to over 80% within 6 h, considerably higher than the tPA release from Chi@tPA without RGD peptides after incubation with activated platelets (about 44% within 6 h). By comparison, when incubated with resting platelets, RGD‐Chi@tPA released only around 42% of the entrapped tPA within 6 h. These results indicate that tPA release from RGD‐Chi@tPA was triggered by their specific interactions with activated platelets as a result of selective binding of RGD peptides on the nanocoacervate surface to *α*_IIb_ *β*_3_ on the activated platelet surface. This was further consolidated by the activated platelet concentration‐dependent tPA release profile displayed in Figure S15, Supporting Information. These findings are consistent with our reported work on the activated‐platelet‐triggered drug release induced by the fibrinogen‐mimicking multifunctional liposomes.^[^ ^33^ ^]^

### 2.3 In Vitro Targeted Fibrinolysis and Thrombolysis

The excellent thrombus‐targeting ability and activated‐platelet‐triggered tPA release profile of RGD‐Chi@tPA prompted the further evaluation of its targeted fibrinolysis and thrombolysis in vitro. First, the selective fibrinolytic activity of RGD‐Chi@tPA in the presence of activated platelets was investigated on fibrin agar plates.^[^ ^31^, ^33^, ^34^ ^]^ The calculated areas of the fibrin clot lysis zone are shown in Figure 2D. It was found that RGD‐Chi@tPA caused significant fibrin lysis and the area of the lysis zone was 1.02 ± 0.15 cm^2^, which was similar to that caused by free tPA (1.14 ± 0.03 cm^2^) but significantly higher than that caused by Chi@tPA (0.35 ± 0.06 cm^2^), RGD‐Chi (0.02 ± 0.02 cm^2^), and PBS buffer (0.01 ± 0.01 cm^2^), respectively. These results suggest that RGD‐Chi@tPA can facilitate efficient fibrin lysis in the presence of activated platelets at a thrombus site. Further, the targeted thrombolysis ability of RGD‐Chi@tPA was evaluated by a halo blood clot assay.^[^ ^33^, ^54^, ^55^ ^]^ As shown in Figure 2E, the thrombolytic activity of RGD‐Chi@tPA was a bit lower than free tPA at the initial time points, which could be ascribed to the time required for tPA release from the nanocoacervates.^[^ ^34^ ^]^ It is interesting to note that after 35 min of treatment, RGD‐Chi@tPA, like free tPA, caused complete thrombus dissolution. By comparison, the degree of thrombolysis after 35 min of treatment reached about 44% for Chi@tPA without RGD, while less than 1% for RGD‐Chi and PBS buffer. This demonstrated that RGD‐Chi@tPA had considerably higher thrombolytic activity compared to Chi@tPA without RGD.

### 2.4 Pharmacokinetics and Biodistribution Assessments

Pharmacokinetic evaluation in Sprague–Dawley (SD) rats was conducted by measuring the FITC‐tPA concentration in plasma after intravenous injection of the FITC‐labeled free tPA, Chi@tPA, and RGD‐Chi@tPA (equivalent tPA dose of 10 mg kg^−1^), respectively. As shown in Figure 2F, the Chi@tPA and RGD‐Chi@tPA nanocoacervates both demonstrated a marked improvement in the retention time compared to free tPA. The circulation half‐lives of Chi@tPA and RGD‐Chi@tPA were ≈64.4 and ≈61.8 min, respectively, significantly longer than that of free tPA at only ≈3.6 min (Figure S16, Supporting Information). The improved pharmacokinetic profiles of the nanocoacervates may be attributed to the creation of a protection layer of chitosan molecules surrounding tPA, thus inhibiting the uptake by macrophages and reticular endothelial systems.^[^ ^56^ ^]^ This data is consistent with the previous report by other researchers that the circulation half‐life of a drug was increased after being entrapped in chitosan‐based nanoparticles.^[^ ^57^ ^]^ The presence of RGD peptides on the RGD‐Chi@tPA surface had no obvious effect on the pharmacokinetic profile as compared with Chi@tPA, which was due to the fact that this work was performed in healthy animals without thrombi. To track the distribution of those different tPA formulations in vivo, the mice were sacrificed at 4 h post‐administration, and the hearts, livers, spleens, lungs, and kidneys were collected. As shown in Figure S17, Supporting Information, tPA was found mainly in the kidney and liver for the Chi@tPA and RGD‐Chi@tPA groups, which indicates that the nanocoacervates could accumulate in metabolic organs. As expected, after injection of RGD‐Chi@tPA, the concentrations of tPA in all major organs were higher than those in the free tPA group, further consolidating that RGD‐Chi@tPA can prolong the retention time in blood compared to free tPA.

### 2.5 Mouse Tail Thrombus Model and Blood Flow Assessment

**Figure 3:**
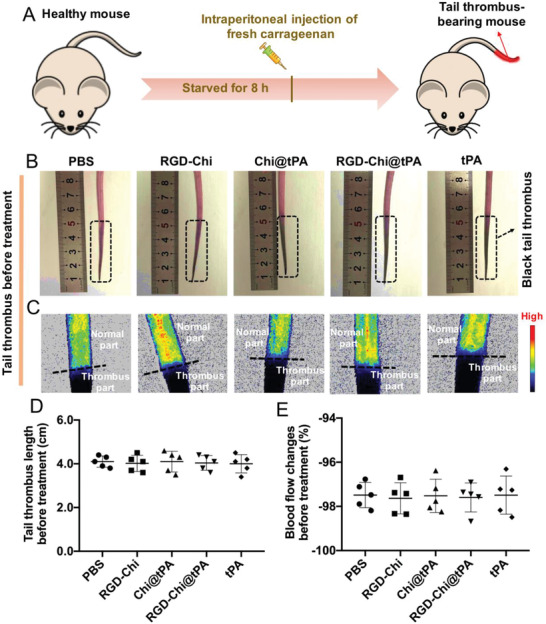
A) Illustration of a KM mouse tail thrombus model established via intraperitoneal injection with fresh carrageenan (20 mg kg^−1^). B) Photographs of the black tail thrombi, C) bloodstream images, and D) lengths of tail thrombus in five mouse groups before treatment with PBS buffer, RGD‐Chi, Chi@tPA, RGD‐Chi@tPA, and free tPA, respectively. E) Blood flow changes of the tail thrombus part relative to the normal part in five mouse groups before treatment with PBS buffer, RGD‐Chi, Chi@tPA, RGD‐Chi@tPA, and free tPA, respectively. Data are presented as the average ± standard deviation (*n* = 5).

A mouse tail thrombus model was set up to evaluate the targeted thrombolytic effect of RGD‐Chi@tPA.^[^ ^18^, ^58^ ^]^ As illustrated in **Figure** **3**A, Kunming (KM) mice were starved for 8 h, followed by treatment with fresh carrageenan (20 mg kg^−1^) via intraperitoneal injection to induce a black tail thrombus.^[^ ^18^ ^]^ Figure 3B shows that before treatment with PBS buffer, RGD‐Chi, Chi@tPA, RGD‐Chi@tPA, and free tPA, respectively, the mice with a similar length of black tail thrombus were randomly divided into five groups. The initial lengths of tail thrombus in all experimental groups were all measured to be ≈4 cm before treatment (Figure 3D). A laser speckle contrast imager (LSCI) was applied to monitor the blood flow after the tail thrombus model was established. As visualized in Figure 3C, the blood flow intensity in the black tail thrombus part (bottom) was significantly lower than that in the normal tail part (top). The blood flow changes relative to the normal tail were measured to estimate the degree of blockage of the bloodstream in the black tail thrombus. Compared to the normal tail part, the blood flow in the black tail thrombus part in all original experimental groups was clearly blocked to a similar degree of ≈97.5% (Figure 3E).

### 2.6 In Vivo Targeted Thrombolysis

**Figure 4:**
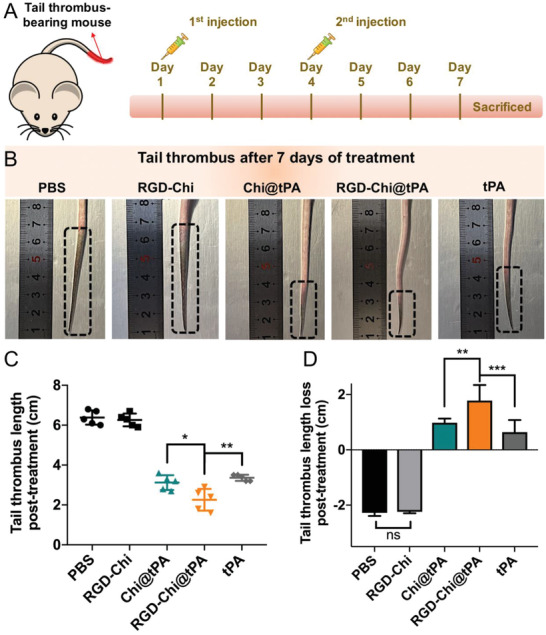
A) The tail thrombus‐bearing KM mice were randomly divided into five groups for intravenous injection with PBS buffer, RGD‐Chi, Chi@tPA, RGD‐Chi@tPA, and free tPA, respectively, on days 1 and 4. For each injection, the tPA dose was 10 mg kg^−1^. B) Photographs of KM mouse tail thrombi, C) tail thrombus lengths, and D) tail thrombus length losses after 7 days of treatment with PBS buffer, RGD‐Chi, Chi@tPA, RGD‐Chi@tPA, and free tPA, respectively. Data are presented as the average ± standard deviation (*n* = 5). Statistical analysis was performed by the ANOVA test (*p < 0.05, **p < 0.01, and ***p < 0.001).

The tail thrombus‐bearing mice were administered with PBS buffer, RGD‐Chi, Chi@tPA, RGD‐Chi@tPA, and free tPA (equivalent tPA dose of 10 mg kg^−1^), respectively, to evaluate their thrombolytic efficacy in vivo (**Figure** **4**A). The tail thrombus length was recorded and its bloodstream recovery was evaluated after 7 days of treatment. It was found that the size of the thrombus in the control groups (PBS buffer and RGD‐Chi) became larger (Figure 4B), with the tail thrombus length being increased to ≈6.3 cm (Figure 4C). As a comparison, after treatment with Chi@tPA, RGD‐Chi@tPA, and free tPA, respectively, thrombus growth was inhibited and some pre‐formed thrombi were dissolved due to thrombolysis by tPA, leading to the significantly decreased tail thrombus length (Figure 4C,D). As shown in Figure 4D, the RGD‐Chi@tPA‐treated group exhibited an effective thrombolytic effect, demonstrating the most significant tail thrombus length loss (≈1.78 cm) as compared to the groups treated with Chi@tPA (≈0.98 cm) or free tPA (≈0.64 cm). The highly efficient thrombolysis induced by RGD‐Chi@tPA was attributed to the targeting of RGD‐Chi@tPA to thrombi (Figure 2A,B) and consequently the efficient triggered tPA release specifically at the thrombus site (Figure 2C).

**Figure 5:**
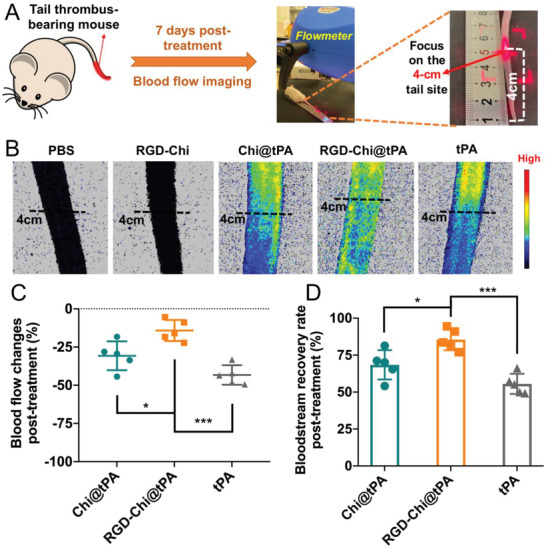
A) The same site at the KM mouse tail length of 4 cm was focused by a laser blood flowmeter to evaluate the bloodstream recovery after 7 days of treatment. B) Bloodstream images, C) blood flow changes in the tail part below the 4‐cm site relative to that above the 4‐cm site, and D) bloodstream recovery rates of the tail thrombus after treatment with PBS buffer, RGD‐Chi, Chi@tPA, RGD‐Chi@tPA, and free tPA (equivalent tPA dose of 10 mg kg^−1^), respectively. Data are presented as the average ± standard deviation (*n* = 5). Statistical analysis was performed by the ANOVA test (*p < 0.05 and ***p < 0.001).

Furthermore, the vascular recanalization of blood vessels after treatment was detected by a laser blood flowmeter. For the purpose of comparisons, the same site at the tail length of 4 cm (the demarcation point between the initial thrombus part of the normal part) was focused by laser (**Figure** **5**A). As shown in Figure 5B, both control groups (PBS buffer and RGD‐Chi) displayed a negligible blood flow intensity in both tail parts above and below the 4‐cm site (due to the increased tail thrombus length shown in Figure 4), indicative of no bloodstream recovery. Interestingly, treatment with RGD‐Chi@tPA for 7 days induced a higher blood flow intensity in the tail part below the 4‐cm site than that treated with Chi@tPA or free tPA. Figure 5C shows that after treatment with RGD‐Chi@tPA, the blood flow changes in the tail part below the 4‐cm site was recovered to a degree of blockage at ≈14% relative to that above the 4‐cm site (the original normal part). This recovery was considerably enhanced than the groups treated with Chi@tPA (degree of blockage at ≈30%) or free tPA (degree of blockage at ≈43%). As shown in Figure 5D, the group treated with RGD‐Chi@tPA displayed a significantly higher bloodstream recovery than that treated with Chi@tPA or free tPA. This further consolidated that RGD‐Chi@tPA could significantly improve the thrombolytic therapy as compared to Chi@tPA and free tPA.

### 2.7 Thrombus Targeting and Penetration of RGD‐Chi@tPA

**Figure 6:**
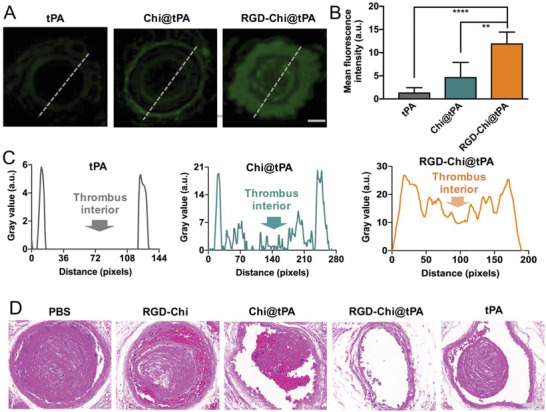
Fluorescence images showing the KM mouse tail thrombus slices which were collected and stained with the FITC‐labeled tPA, Chi@tPA, and RGD‐Chi@tPA (equivalent tPA dose of 2.5 µg mL^−1^), respectively, at 37 °C for 35 min (scale bar: 100 µm). B) Mean fluorescence intensity of FITC in the fluorescence images of tail thrombi post staining as analyzed by Fiji. Data are presented as the average ± standard deviation (*n* = 5). Statistical analysis was performed by the ANOVA test (*p < 0.05 and ****p < 0.0001). Dotted line indicates the cross‐section used to produce the intensity profiles shown in (C) with the use of Fiji. D) Representative H&E staining photomicrographs of the slices of tail thrombi of KM mice after 7 days of treatment with PBS buffer, RGD‐Chi, Chi@tPA, RGD‐Chi@tPA, and free tPA (equivalent tPA dose of 10 mg kg^−1^), respectively (scale bar: 100 µm).

In order to better understand the enhanced thrombolysis by RGD‐Chi@tPA in the carrageenan‐induced mouse tail thrombus model, its thrombus targeting and penetration ability were investigated by fluorescence imaging and histochemical assay. First, the mouse tail thrombus slices were collected and stained with the FITC‐labeled free tPA, Chi@tPA, and RGD‐Chi@tPA (equivalent tPA dose of 2.5 µg mL^−1^), respectively, at 37 °C for 35 min, and then washed with PBS buffer three times under shaking before fluorescence microscopy. As shown in **Figure** **6**A, after staining of the tail thrombus with the FITC‐labeled RGD‐Chi@tPA, strong green fluorescence was detected in the thrombus, indicative of its effective thrombus targeting due to the selective binding of RGD on the nanoceacervate surface to *α*_IIb_ *β*_3_ on the activated platelet surface at a thrombus site. However, only weak green signals were detected for the tail thrombi stained with Chi@tPA or free tPA without the thrombus targeting capacity (Figure 6A,B). Figure 6C shows that for the free tPA‐treated group, very weak fluorescence was observed only on the thrombus edge, indicative of the low affinity to a thrombus and its difficulty to penetrate the vascular structure and transport to the thrombus interior. Chi@tPA displayed the enhanced green fluorescence on the thrombus edge, while with a weak signal inside the thrombus (Figure 6A,C). This improvement over free tPA, although still not ideal for thrombolytic therapy, might be due to the enhanced penetration of nano‐sized particles and electrostatic attraction between positively charged chitosan on the nanoceacervate surface and negatively charged thrombus components.^[^ ^27^, ^28^ ^]^ It is interesting to note that staining with RGD‐Chi@tPA resulted in remarkably stronger green fluorescence across the thrombus from the edge to the core as compared to Chi@tPA without RGD (Figure 6A,C). This was attributed to the effective thrombus targeting of RGD‐Chi@tPA due to the selective binding through the ligand‐receptor interactions, which in turn could facilitate the penetration of RGD‐Chi@tPA to the thrombus interior and trigger the release of tPA therein. Therefore, as compared to free tPA, the improvement of thrombus penetration by the RGD‐Chi@tPA nanocoacervates could be attributed to three aspects. First, the nanocoacervates have a controllable size (259.3 ± 5.2 nm) within the reported range of ≈250–320 nm suitable for penetration in the porous structure of fibrin gels and blood clots.^[^ ^44^, ^45^ ^]^ Second, the positive charges resulting from chitosan on the RGD‐Chi@tPA surface could facilitate deeper and more efficient thrombus penetration through electrostatic adsorption to the negatively charged compositions inside a thrombus.^[^ ^27^ ^]^ Finally, the thrombus targeting of RGD‐Chi@tPA could further improve thrombus penetration through the interactions between RGD ligands on the nanoparticles surface and *α*_IIb_ *β*_3_ integrins on the activated platelet surface inside a thrombus.^[^ ^28^ ^]^

Furthermore, the significantly improved thrombolytic therapy in the mouse tail thrombus model was proven to result from the efficient thrombus targeting and penetration through hematoxylin‐eosin (H&E) staining of the slices of tail thrombi of mice after 7 days of in vivo treatment with PBS buffer, RGD‐Chi, Chi@tPA, RGD‐Chi@tPA, and free tPA (equivalent tPA dose of 10 mg kg^−1^), respectively. As shown in Figure 6D, a plugged thrombus was clearly retained in the control groups (PBS buffer and RGD‐Chi). Treatment with Chi@tPA or free tPA caused a decrease in thrombus size. It was observed that the retained smaller thrombus after the free tPA treatment appeared to have a smooth surface, which resulted from the poor thrombus penetration (Figure 6A,C) and the consequent thrombolytic activity limited on the thrombus surface. However, the retained smaller thrombus after treatment with Chi@tPA was fragmentary as a result of a multi‐faceted dissolution of the thrombus due to the slightly improved thrombus penetration of the Chi@tPA nanoceacervate as compared to free tPA (Figure 6A,C). As a comparison, treatment with RGD‐Chi@tPA led to the complete thrombus dissolution and unclogging of the blood vessel, further confirming its substantially higher thrombolytic efficacy than Chi@tPA and free tPA.

### 2.8 In Vivo Biosafety Profiles

**Figure 7:**
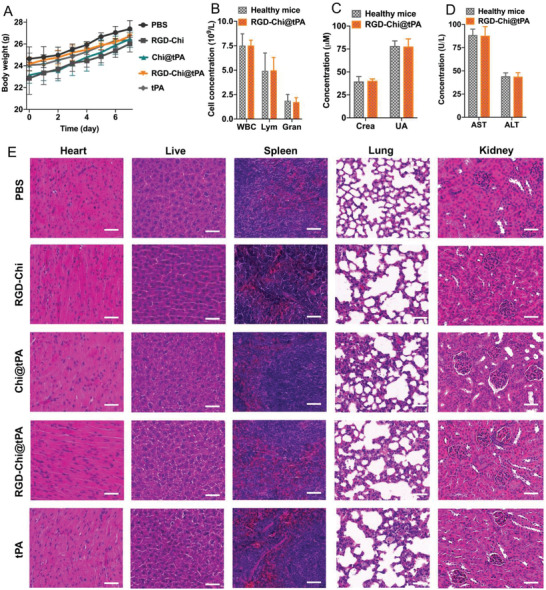
A) Body weights of KM mice after 7 days of treatment with PBS buffer, RGD‐Chi, Chi@tPA, RGD‐Chi@tPA, and free tPA (equivalent tPA dose of 10 mg kg^−1^), respectively. Data are presented as the average ± standard deviation (*n* = 5). B) Hematology studies of inflammatory cells (WBC, white blood cells; Lym, lymphocytes; Gran, granulocytes) in blood, C) biochemical indexes of creatinine (Crea) and uric acid (UA) in serum, and D) biochemical indexes of aspartate aminotransferase (AST) and alanine aminotransferase (ALT) in serum of KM mice containing thrombi in their tails after 7 days of treatment with RGD‐Chi@tPA (healthy KM mice as control). Data are presented as the average ± standard deviation (*n* = 3). E) Representative H&E staining photomicrographs of major organs (heart, liver, spleen, lung, and kidney) after 7 days of treatment with PBS buffer, RGD‐Chi, Chi@tPA, RGD‐Chi@tPA, and free tPA, respectively (scale bar: 100 µm).

The body weight of mice was monitored, blood was collected for hematology and biochemical analyzes, and main organs were dissected for H&E staining post‐treatment to assess potential unwanted side effects. As shown in **Figure** **7**A, in all the treatment groups (PBS buffer, RGD‐Chi, Chi@tPA, RGD‐Chi@tPA, and free tPA), no noticeable body weight loss was detected in 1 week. The hematology results demonstrated that treatment with RGD‐Chi@tPA caused negligible changes to the blood indexes including inflammatory cells (white blood cells: WBC, lymphocytes: Lym, and granulocytes: Gran) (Figure 7B) and other main indexes (lymphocytes percentage: %Lym, granulocytes percentage: %Gran, red blood cell: RBC, platelet: PLT, hemoglobin: HGB, platelet distribution width: PDW, hematocrit value: HCT, thrombocytocrit: PCT, coefficient of variation of red blood cell volume distribution width: RDW‐CV, standard deviation of red blood cell volume distribution width: RDW‐SD, mean platelet volume: MPV, mean corpuscular volume: MCV, platelet large cell count: P‐LCC, and platelet large cell ratio: P‐LCR) (Figure S18A–N, Supporting Information). Considering that RGD‐Chi@tPA mainly accumulated in the kidney and liver after injection (Figure S17, Supporting Information), the main biochemical indexes of kidney (creatinine: Crea, uric acid: UA, and Urea) and liver (aspartate aminotransferase: AST, alanine aminotransferase: ALT, and alkaline phosphatase: ALP) were tested. As shown in Figure 7C,D, and Figure S18O,P, Supporting Information, all those main biochemical indexes remained to be similar to the normal level measured in healthy mice. In addition, the H&E staining shown in Figure 7E demonstrated that the RGD‐Chi@tPA‐treated group, like the other treatment groups (PBS buffer, RGD‐Chi, Chi@tPA, and free tPA), did not present noticeable toxicity or inflammatory cells in major organs (heart, liver, spleen, lung, and kidney). These results suggest that RGD‐Chi@tPA is biologically safe in vivo.

**Figure 8:**
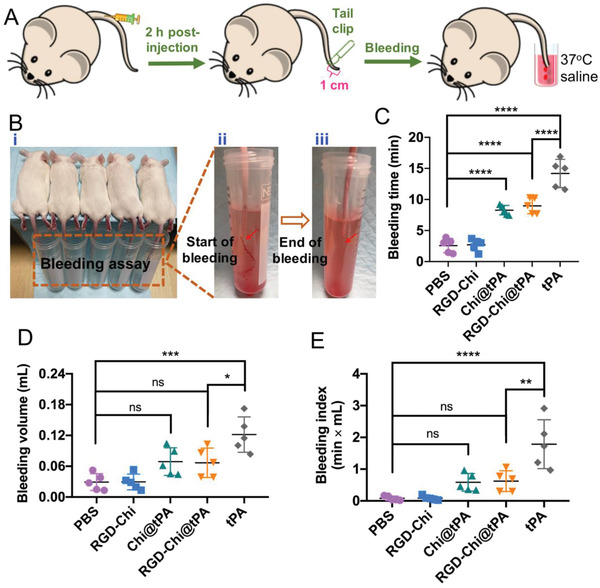
A) Schematic illustration of a tail bleeding assay using KM mice. B) i) Bleeding assay was performed by amputation of the tail tip, immediately followed by submergence into a 37 °C saline solution; ii) Photograph showing the start of bleeding in the assay; iii) Photograph showing the end of bleeding in the assay. C) Bleeding time, D) bleeding volume, and E) bleeding index in the tail‐cut mice after injection with PBS buffer, RGD‐Chi, Chi@tPA, RGD‐Chi@tPA, and free tPA, respectively. The bleeding index was defined by the product of bleeding time (min) and bleeding volume (mL). Data are presented as the average ± standard deviation (*n* = 5). Statistical analysis was performed by the ANOVA test. (*p < 0.05, **p < 0.01, ***p < 0.001, ****p < 0.0001, and ns, not significant).

Furthermore, a tail bleeding assay was used to assess the hemorrhagic risk of RGD‐Chi@tPA (**Figure** **8**A). In this assay, a longer tail bleeding time and a larger bleeding volume represent a higher hemorrhagic risk.^[^ ^59^, ^60^ ^]^ As shown in Figures 8A and 8B, a distal 1‐cm segment of the tail was amputated with a scalpel to induce bleeding, and the bleeding time and volume were recorded at 2 h post‐injection of PBS buffer, RGD‐Chi, Chi@tPA, RGD‐Chi@tPA, and free tPA, respectively. Figure 8C shows that in the mice injected with PBS buffer or RGD‐Chi, complete blood coagulation occurred ≈3 min after bleeding due to the intrinsic hemostatic functions of the blood system. The bleeding time post‐injection with Chi@tPA or RGD‐Chi@tPA was prolonged to ≈8 min, whilst significantly shorter compared to injection by free tPA (≈14 min). It was found that the bleeding volume induced by free tPA was increased notably as compared to that induced by Chi@tPA or RGD‐Chi@tPA (Figure 8D). Taking into account the significance of both bleeding time and volume in the assay, we calculated the bleeding index to evaluate hemorrhagic risk, as defined by the product of bleeding time and bleeding volume.^[^ ^61^ ^]^ As shown in Figure 8E, the bleeding index in mice treated with free tPA was about 1.8, which was significantly larger than that in the RGD‐Chi@tPA‐treated group (≈0.6). Those results suggest that encapsulation of tPA in RGD‐Chi@tPA could remarkably reduce the nonspecific hemorrhagic side effect of free tPA in thrombolytic therapy.

## 3 Conclusions

In summary, a fibrinogen‐mimicking, activated‐platelet‐sensitive nanocoacervate, namely RGD‐Chi@tPA, was successfully constructed by a “one‐pot” coacervation process through electrostatic interactions and characterized for enhanced targeted thrombolytic therapy. RGD‐Chi@tPA had a small particle size with narrow size distribution. Flow cytometry and CLSM measurements showed targeting of RGD‐Chi@tPA to activated platelets at a thrombus site. An activated‐platelet‐sensitive release of tPA from RGD‐Chi@tPA was achieved and its in vitro targeted fibrinolytic and thrombolytic activities were demonstrated in the fibrin clot and halo blood clot models. In a carrageenan‐induced mouse tail thrombus model, the efficient thrombus targeting and penetration of RGD‐Chi@tPA enabled the significantly enhanced thrombolytic therapy as compared to free tPA. The in vivo biosafety studies proved that RGD‐Chi@tPA had excellent biocompatibility and a minimal unwanted hemorrhagic risk. This “all‐in‐one” biomimetic nanomedicine represents a promising strategy for safe and effective targeted thrombolytic therapy.

## 4 Experimental Section

### Materials

Chitosan (medium molecular weight), *N*‐hydroxysulfosuccinimide sodium salt (Sulfo‐NHS), 1‐(3‐dimethylaminopropyl)‐3‐ethyl carbodiimide hydrochloride (EDC), tripeptide arginine‐glycine‐aspartic acid (RGD), FITC, bovine serum albumin (BSA), thrombin, plasminogen, fibrinogen (Fg), acid citrate dextrose (ACD), agar, tri(hydroxymethyl)aminomethane, Dulbecco's phosphate‐buffered saline (D‐PBS), sodium chloride (NaCl), magnesium chloride (MgCl_2_), calcium chloride (CaCl_2_), Triton X‐100, dichloromethane, methanol, the BCA assay kit, and the tPA chromogenic activity assay kit S‐2251 were purchased from Sigma‐Aldrich (Dorset, UK). tPA was a product of Boehringer Ingelheim (Germany). Dialysis tubes were purchased from Fisher Scientific (Loughborough, UK). Whole sheep blood in ACD and defibrinated red blood cells (RBCs) were obtained from TCS Biosciences Ltd. (Buckingham, UK). Acetic acid, carrageenan, hydrochloric acid, sodium hydroxide, dimethyl sulfoxide (DMSO), and absolute ethyl alcohol were obtained from Adamas‐beta (Shanghai, China). The deuterium oxide (D_2_O) and deuterated dimethyl sulfoxide (DMSO‐*d*_6_) were purchased from VWR (Lutterworth, UK).

### RGD‐Chi Conjugate Synthesis and Characterization

Chitosan solution (1.0%, w/v) was prepared by dissolving chitosan into aqueous acetic acid (2.0%, v/v). Then, 150 µL of EDC aqueous solution (1.0 wt%) and 150 µL of sulfo‐NHS aqueous solution (2.5 wt%) were added to a reaction flask filled with 5 mL of RGD aqueous solution (10 mg mL^−1^) and magnetically stirred for 30 min at 4 °C in an ice bath. Under continuous stirring, the chitosan solution was added to the flask at a 1.0:0.1 molar ratio (chitosan:RGD) to prepare the RGD‐grafted chitosan conjugate (RGD‐Chi). The reaction mixture was stirred at room temperature for 3 h. The synthesized RGD‐Chi was purified by dialysis against distilled water for 48 h. The content percentage of RGD was measured by the BCA kit. The final product of RGD‐Chi was characterized by a Spectrum 100 FTIR spectrometer (PerkinElmer, USA) and a ^1^H NMR spectrometer (Bruker, Germany) in 1% v/v acetic acid included D_2_O at room temperature.

### RGD‐Chi@tPA Synthesis and Characterization

Chi (0.02 mm) and RGD‐Chi (0.02 mm) solutions were prepared in aqueous acetic acid (20.0%, v/v), and tPA stock solution (0.015 mm) was prepared in PBS buffer (pH 7.4). Nanocoacervates were prepared based on electrostatic interactions of cationic Chi (or RGD‐Chi) and anionic tPA. Briefly, a predetermined tPA solution was added dropwise into Chi (or RGD‐Chi) solution at different molar ratios at 4 °C and nanocoacervates were spontaneously formed under constant stirring at 4 °C for 1 h. The final nanocoacervate aqueous solution was obtained by dialysis against PBS buffer. The hydrodynamic size, PDI, morphology, and zeta potential of the nanocoacervates were measured by DLS (Malvern Zetasizer Nano S, UK), SEM (JEOL JSM‐5610LV, Japan), and zeta potential analysis (Brookhaven ZetaPALS analyzer, USA), respectively. The amounts of tPA loaded in the nanocoacervates were determined by the chromogenic substrate S‐2251 assay. The CE and EE were defined and calculated by the following Equations (1) and (2), respectively.

(1) $$\mathrm{Coacervation}\;\mathrm{efficiency}\left(\%\right)=\frac{m_{w}}{m_{T}}\times 100$$

where *m*_w_ is the final weight of nanocoacervates and *m*_T_ is the total initial weight of starting materials in the coacervation solution.

(2) $$\mathrm{Encapsulation}\;\mathrm{efficiency}\left(\%\right)=\frac{m_{l}}{m_{t}}\times 100$$

where *m_l_* is the weight of loaded tPA in the nanocoacervates and *m_t_* is the total initial weight of tPA in the coacervation solution.

### Animal Experiments

All animal experimental procedures were performed in agreement with the guidelines and ethical approval was obtained from the Animal Research Committee of Shanghai Jiao Tong University Affiliated Sixth People's Hospital (approval number: 2021‐0774). Healthy male SD rats (≈220 g) and KM mice (≈22 g) were purchased from Shanghai SLAC Laboratory Animal Co., Ltd.

### Pharmacokinetic and Biodistribution Studies

SD rats were anesthetized with sodium pentobarbital and randomly divided into three groups (*n* = 3). The FITC‐labeled free tPA, Chi@tPA, and RGD‐Chi@tPA solutions were intravenously injected via tail vein at the equivalent tPA dose of 10 mg kg^−1^. After treatment, ≈0.3–0.4 mL of blood was drawn from the eye socket at 1, 3, 5, 8, 10, 15, and 30 min for the free tPA group, while at 1, 15, 30, 60, 90, 120, 180, and 240 min for the nanocoacervate formulation groups. At 4 h post‐administration, all mice were sacrificed. The hearts, livers, spleens, lungs, and kidneys were then collected. For plasma samples: plasma was separated by centrifuging the blood samples at 3500 × g for 10 min. 200 µL of plasma was taken for fluorescence measurements by a plate reader (BioTek Epoch, USA). For tissue samples: all tissues were rinsed in saline, dried using a paper towel carefully, and finally homogenized. 200 µL of tissue sample was taken for fluorescence measurements by the plate reader. The concentrations of tPA in the blood or tissue samples were calculated by using the standard curve. Half‐lives were calculated according to the following equation:

(3) $$t_{1/2}=\frac{0.963}{K_{e}}$$

where *K*_e_ is the elimination rate constant.

### Mouse Tail Thrombus Model

KM mice with tails longer than 10 cm were starved for 8 h and treated with 1.5% w/v fresh carrageenan saline solution (20 mg kg^−1^) via intraperitoneal injection, and then the mice were fed at 20 °C. After about 24 h, the black tail thrombus could be observed.

### Measurement of Blood Flow

Tail thrombus‐bearing KM mice were anesthetized and the average blood flow intensity in the tail was assessed by an LSCI with a PeriCam PSI HR system (Perimed, Sweden). Images of the bloodstream were collected and the average blood flow intensity in the regions of interest (ROI) was analyzed by PIMSoft software. The blood flow changes and bloodstream recovery rate were calculated according to the following equations:

(4) $$\mathrm{Blood}\;\mathrm{flow}\;\mathrm{changes}\left(\%\right)=\frac{{\mathrm{Flow}}_{t}-{\mathrm{Flow}}_{n}}{{\mathrm{Flow}}_{n}}\times 100$$

where Flow_t_ is the blood flow intensity in the thrombus part and Flow_n_ is the blood flow intensity in the normal tail part.

(5) $$\mathrm{Bloodstream}\;\mathrm{recovery}\;\mathrm{rate}\left(\%\right)=\frac{{\mathrm{FC}}_{\mathrm{before}}-{\mathrm{FC}}_{\mathrm{after}}}{{\mathrm{FC}}_{\mathrm{before}}}\times 100$$

where FC_before_ is the blood flow changes in tail thrombus before treatment and FC_after_ is blood flow changes in tail thrombus after treatment.

### Thrombus Penetration

The tails with thrombi of KM mice were separated and stored in 4% paraformaldehyde solution at 4 °C for 48 h. The tails were then decalcified and embedded in paraffin and cut into small slices. The resulting slices were stained with the FITC‐labeled free tPA, Chi@tPA, and RGD‐Chi@tPA (equivalent tPA dose of 2.5 µg mL^−1^), respectively, at 37 °C for 35 min. Subsequently, the slices were washed with PBS buffer three times under shaking (5 min per time) and finally mounted and observed with an ECLIPSE 80i fluorescence microscope (Nikon, Japan).

### In Vivo Thrombolysis

KM mice with a similar length of black tail thrombus were divided randomly into five groups and intravenously injected with PBS buffer, RGD‐Chi, Chi@tPA, RGD‐Chi@tPA, and free tPA (equivalent tPA dose of 10 mg kg^−1^). The length of the tail thrombus was recorded before and after each treatment. In addition, the body weight of each mouse was monitored every day. After 7 days of observation, all mice were sacrificed and their tails were separated and stored in 4% paraformaldehyde solution at 4 °C for 48 h. The tails were then decalcified and embedded in paraffin and cut off into small slices. The slices were finally stained with H&E for assessment of thrombolysis by histochemical analysis.

### In Vivo Biosafety Assessments

After 7 days of treatment with PBS buffer, RGD‐Chi, Chi@tPA, RGD‐Chi@tPA, and free tPA (equivalent tPA dose of 10 mg kg^−1^), respectively, KM mice were sacrificed, the blood was collected and major organs (hearts, livers, spleens, lungs, and kidneys) were separated. The hematology data was measured by a BC‐30Vet automatic blood cell analyzer (Mindray, China). The serum biochemistry was assessed by a Chemray 240 automatic chemistry analyzer (Rayto, China). The major organs were embedded in paraffin and small slices were cut off for H&E staining for biosafety evaluation.

### Tail Bleeding Assay

Healthy KM mice were divided randomly into five groups (*n* = 5) and intravenously injected with PBS buffer, RGD‐Chi, Chi@tPA, RGD‐Chi@tPA, and free tPA (equivalent tPA dose of 10 mg kg^−1^), respectively. At 2 h post‐injection, the mice were anesthetized and placed in prone position. The distal 1‐cm section of the tail was cut off with a scalpel and then submerged into the pre‐warmed saline solution at 37 °C. Mice were observed for 25 min. The bleeding time was defined as the time for the transected tail to stop bleeding for at least 10 s. To calculate the bleeding volume, blood cells were centrifuged at 4000 g for 10 min. Cell pellets were resuspended in 1 mL of 1% Triton X‐100 and the bleeding volume was measured according to the hemoglobin concentrations compared to the standard curve.

### Statistical Analysis

Results were shown as the average ± standard deviation (*n* ≥ 3) and graphed by using GraphPad Prism (version 8.0). Differences between two groups were analyzed for significance by using an unpaired Student's *t*‐test and comparisons among multiple groups were assessed for significance using the one‐way analysis of variance (ANOVA) test. Differences were considered to be statistically significant when the *p*‐value was <0.05.

## Conflict of Interest

The authors declare no conflict of interest.

## Supporting information

Supporting Information

## Data Availability

The data that support the findings of this study are openly available in Zenodo at https://doi.org/10.5281/zenodo.5830242, reference number 5830242.
