# Supplementary material for: A Fibrinogen‐Mimicking, Activated‐Platelet‐Sensitive Nanocoacervate Enhances Thrombus Targeting and Penetration of Tissue Plasminogen Activator for Effective Thrombolytic Therapy
Source: Adv Healthc Mater. 2022 Jul 31;11(19):2201265. doi: 10.1002/adhm.202201265 (PMC11468879; doi:10.1002/adhm.202201265)
Supplement: Supplementary file 1 — Supporting Information [file ADHM-11-2201265-s001.pdf]

ADVANCED HEALTHCARE MATERIALS

Supporting Information

for *Adv. Healthcare Mater.*, DOI 10.1002/adhm.202201265

A Fibrinogen-Mimicking, Activated-Platelet-Sensitive Nanocoacervate Enhances Thrombus Targeting and Penetration of Tissue Plasminogen Activator for Effective Thrombolytic Therapy

Yu Huang, Jingxuan Jiang, Jie Ren, Yuanyuan Guo, Qianqian Zhao, Jia Zhou, Yuehua Li* and Rongjun Chen**

Supporting Information

A fibrinogen-mimicking, activated-platelet-sensitive nanocoacervate enhances thrombus targeting and penetration of tissue plasminogen activator for effective thrombolytic therapy

Yu Huang,[a,b,*] Jingxuan Jiang,[a] Jie Ren,[b] Yuanyuan Guo,[a] Qianqian Zhao,[a] Jia Zhou,[a] Yuehua Li,[a,*] and Rongjun Chen[b,*]

[a] Department of Radiology, Shanghai Jiao Tong University Affiliated Sixth People's Hospital, 600 Yi Shan Road, Shanghai, 200233, P. R. China.

[b] Department of Chemical Engineering, Imperial College London, South Kensington Campus, London, SW7 2AZ, United Kingdom.

*Corresponding authors:

Dr. Rongjun Chen. Email: rongjun.chen@imperial.ac.uk

Dr. Yu Huang. Email: yuhuang6y@163.com

Prof. Yuehua Li. Email: liyuehua77@sjtu.edu.cn

1. Supporting experimental methods

1.1 Hemocompatibility assay

Sample solutions of Chi, RGD-Chi, Chi@tPA or RGD-Chi@tPA at different concentrations were prepared in PBS buffer (pH 7.4). Defibrinated red blood cells (RBCs) were washed at least three times with PBS buffer and then resuspended with samples. Negative and positive controls were prepared by resuspending RBCs in buffer alone and deionized water, respectively.[1] The mixtures were incubated in a shaking water bath at 37 °C for 1 h and then centrifuged at 4000 rpm for 4 min. The absorbance of the supernatant at 540 nm was measured using a UV-vis spectrophotometer (Thermo Scientific, USA). The hemolysis percentages were calculated by the following equation:

$$\text{Hemolysis } (\%) = \frac{A_s - A_n}{A_p - A_n} \times 100 \qquad (1)$$

where A_s is the absorbance after incubation with samples; A_n is the absorbance of negative control; A_p is the absorbance of positive control.

1.2 Platelet aggregation assay

Platelet suspension was pre-incubated with RGD-Chi@tPA at the specific tPA dose at 37°C for 30 min. Platelets (2.5×10^8 mL^{-1}) were then added to a 96-well plate (VWR, UK) in the absence or presence of thrombin (1 U mL^{-1}) as an agonist, and the aggregation was measured at 20-s intervals for 15 min using a Power Wave X5 plate reader (Bio-TEK, UK). The temperature was maintained at 37°C throughout the experiment, with a shaking for 7 s before each measurement. Maximum aggregation within 15 min was calculated as a percentage change in absorbance compared to the baseline.

1.3 Binding affinity with activated platelets measured by flow cytometry

Fresh platelets were purified from whole blood with anti-coagulant acid citrate dextrose (ACD) by centrifugation. Briefly, the blood was added into a 2-mL tube and centrifuged at 200 × g for 15 min at 25 ^{o}C to prepare platelet-rich plasma (PRP), and was then washed twice and centrifuged at 800 × g for 15 min at 25 ^{o}C, with 2 μM prostaglandin E1 as an inhibitor of

platelet aggregation. The obtained fresh platelets were resuspended with PBS buffer to the desired concentration.

2 mL of platelets (1.0×10^8 mL^{-1}) were seeded in 6-well plates and activated by incubation with 100 μL of thrombin (1 U mL^{-1}) for at least 20 min. Inactivated (resting) platelets or thrombin-activated platelets were incubated with the FITC-labeled nanocoacervates (equivalent tPA concentration of 0.2 mg mL^{-1}). Free nanocoacervates, which were not attached to platelets, were removed by centrifugation. Data for 1.0×10^4 gated events were collected and detected by using a BD Fortessa II flow cytometer.

1.4 Binding affinity with activated platelets visualized by confocal laser scanning microscopy

2 mL of platelets (1.0×10^8 mL^{-1}) were seeded in 6-well plates, with a collagen-coated glass coverslip on the bottom of each well. After 30 min, 100 μL of thrombin (1 U mL^{-1}) was added onto the platelet-adhered coverslips to ensure activation of platelets after at least 20 min. Inactivated (resting) platelets or thrombin-activated platelets were incubated with the FITC-labeled nanocoacervates (equivalent tPA concentration of 0.2 mg mL^{-1}). Platelets were then fixed with 4.0% formaldehyde for 30 min, followed by rinsing with pH 7.4 PBS buffer three times. Subsequently, the resulting slides were mounted and observed with a Leica SP5 MP confocal microscope.

1.5 *In vitro* tPA release

The tPA release study was performed in the presence of activated platelets to confirm that tPA was released specifically upon interaction with activated platelets.[2-4] 200 μL of platelets (1.0×10^8 mL^{-1}) were placed into a collagen-coated 96-well microplate and activated by treatment with 20 μL of thrombin (1 μM) for at least 20 min. Inactivated (resting) platelets or thrombin-activated platelets were incubated with nanocoacervates (equivalent tPA dose of 0.5 mg mL^{-1}). The amount of released tPA in each well was determined by measuring the absorbance at 405 nm with a GloMax-Multi Microplate Multimode Reader (Promega, USA), according to the

chromogenic substrate S-2251 assay. The percentage of tPA release was calculated according to the following equation:

$$\text{tPA release (\%)} = \frac{A_s - A_n}{A_p - A_n} \times 100 \qquad (2)$$

where A_s is the absorbance after incubation with samples; A_n is the absorbance after incubation with PBS buffer only (negative control); A_p is the absorbance of tPA solution containing the same amount of tPA initially loaded in the nanocoacervates (positive control).

1.6 Fibrin agar plate assay for determination of selective fibrinolytic activity

Fibrin clot lysis by the nanocoacervates was measured by an agar plate assay.[3-5] Briefly, 300 mg agar was dissolved in a buffer mixture (15 mL of 0.05 M Tris-HCl buffer at pH 7.2 and 5 mL of 0.025 M $CaCl_2$ solution). 50 mg fibrinogen was dissolved in 10 mL of Tris-HCl buffer (0.05 M, pH 7.2). The agar solution was mixed with the fibrinogen solution, and then 10 μL of thrombin (4.0 μM) was added under stirring for about 1-3 min. The resulting mixture was spread carefully on a transparent plastic plate and homogeneous gels were obtained at 37 °C after 3 h. Sample wells were created in the plate and 5 μL of plasminogen solution (1 mg mL^{-1}) was then added into each sample well. Before addition samples into the well, nanocoacervates (equivalent tPA dose of 1 mg mL^{-1}) were first treated with activated platelets to induce tPA release. Finally, various samples were added into respective sample wells, respectively, and incubated at 37 °C for 24 h. The area of the lysed zone in each well was measured to evaluate fibrin clot lysis.

1.7 Halo blood clot assay for determination of selective thrombolytic activity

Briefly, a clotting mixture (5 mL of buffer containing 66 mM Tris-HCl, 130 mM NaCl and 45 mM $CaCl_2$; 5μL of 1 μM thrombin, pH 7.4) was freshly prepared. In a 96-well microplate, 5 μL of this clotting mixture was placed on one side of the well bottom, then 15 μL of whole blood was added on the opposite side of the well bottom. Clotting was initiated by mixing the two drops with a pipette tip in a same circular motion to form a homogenous halo shape of

blood around the edge of the well bottom, leaving the center area empty.[3,6,7] The plate was covered and incubated at 37 °C for 30 min. After clot formation, 80 μL of PBS buffer, RGD-Chi, Chi@tPA, RGD-Chi@tPA and free tPA (equivalent tPA dose of 1 mg mL^{-1}) were added simultaneously into respective wells containing halo clots. The dissolution of the halo clots at the indicated time point was determined with a GloMax-Multi Microplate Multimode Reader (Promega, USA) by measuring absorbance at 495 nm caused by RBCs progressively covering the center of the well after clot degradation at 37 °C. The negative control was obtained by adding 80 μL PBS buffer to halo thrombi (without tPA), and the positive control was obtained by mixing 15 μL blood and 85 μL of PBS buffer in a well. The percentage of clot dissolution was calculated according to the following equation:

$$\text{Clot lysis (\%)} = \frac{A_s - A_n}{A_p - A_n} \times 100 \qquad (3)$$

where A_s is the absorbance of the sample well after treatment; A_n is the absorbance of negative control well; A_p is the absorbance of positive control well.

2. Supporting figures

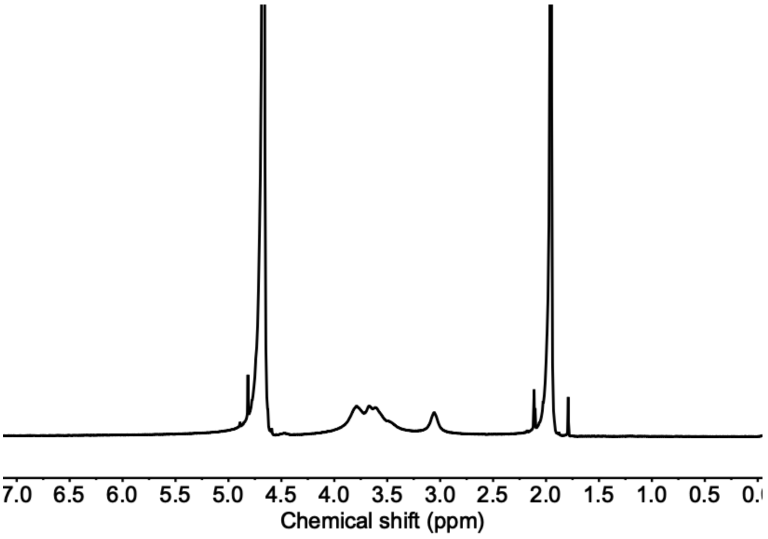

Figure S1. ^{1}H NMR spectrum of chitosan in 1% (v/v) acetic acid included D_2O (400 MHz, 25 °C).

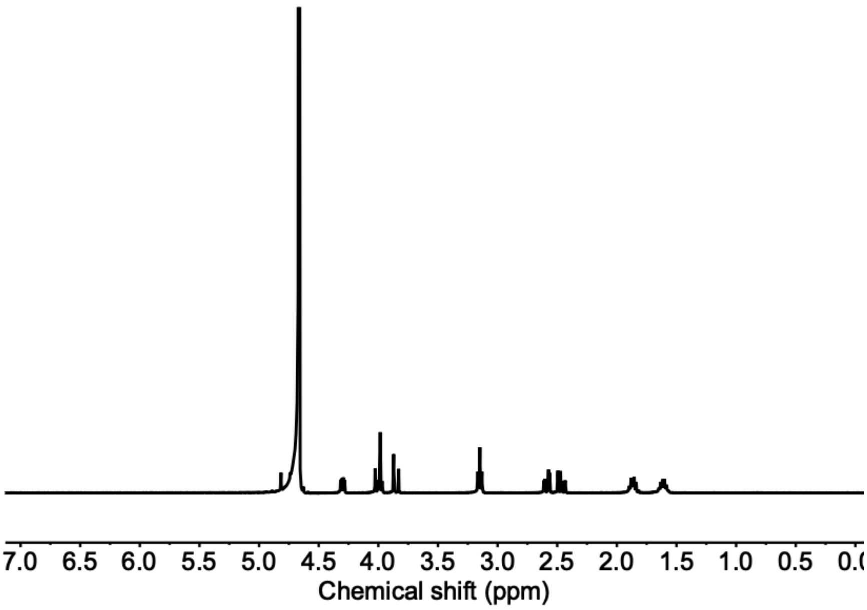

Figure S2. ^{1}H NMR spectrum of RGD in D_2O (400 MHz, 25 °C).

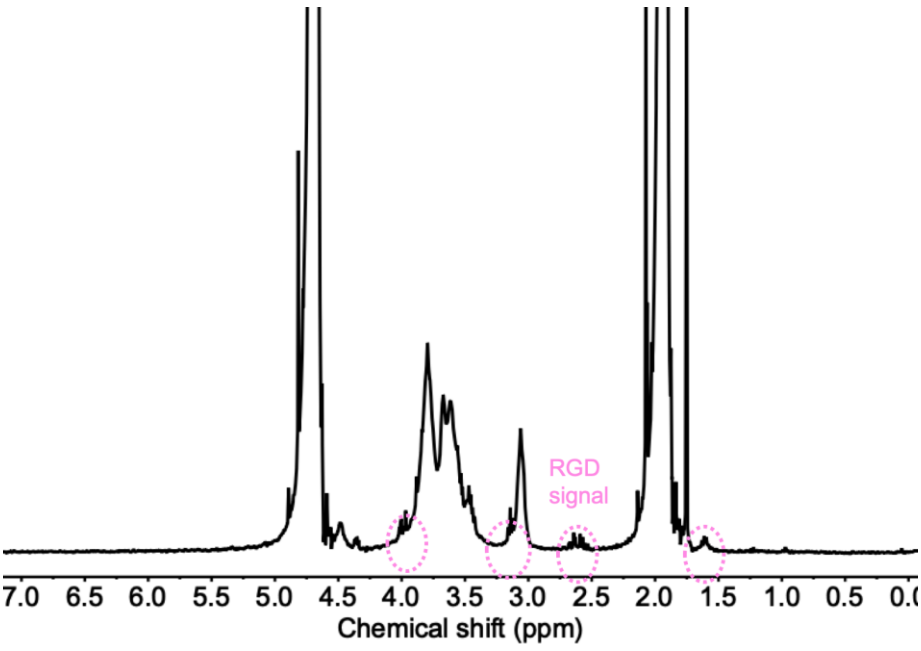

Figure S3. ^{1}H NMR spectrum of RGD-Chi in 1% (v/v) acetic acid included D_2O, (400 MHz, 25 °C).

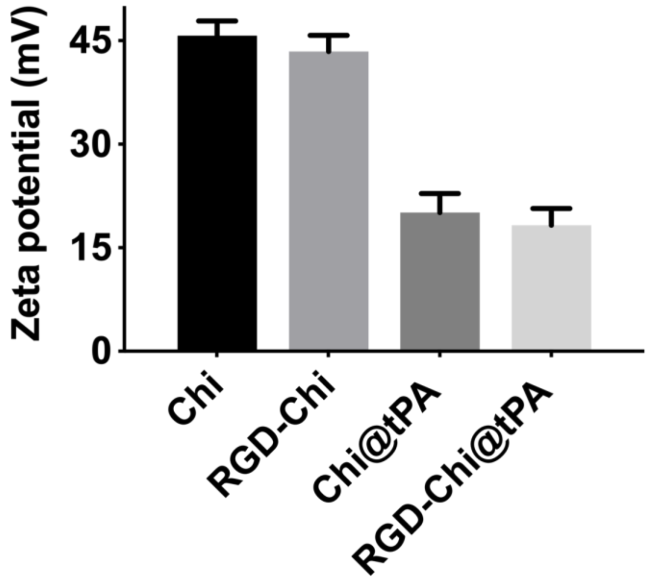

Figure S4. Zeta potential measurements of Chi, RGD-Chi, Chi@tPA and RGD-Chi@tPA (pH 7.4, 25 °C). Data are presented as the average ± standard deviation (n = 3).

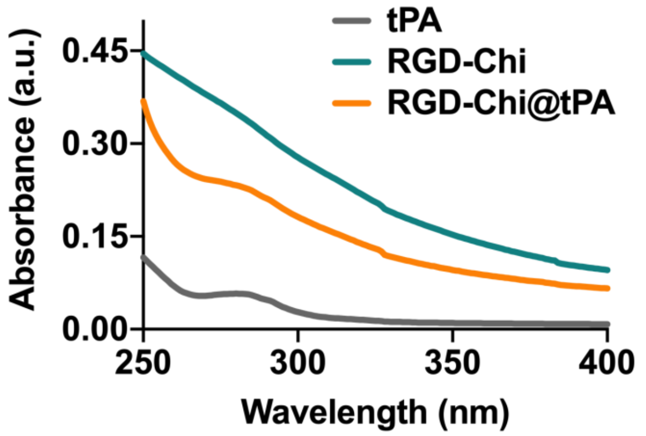

Figure S5. UV-Vis absorption spectra of RGD-Chi (pH 3.5, 25 °C), as well as tPA and RGD-Chi@tPA (pH 7.4, 25 °C).

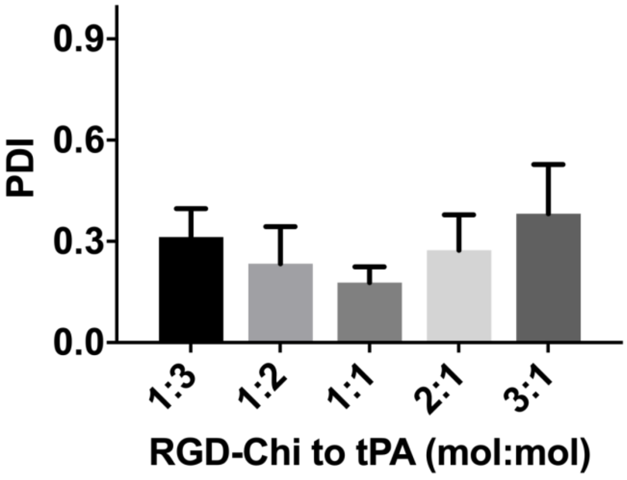

Figure S6. The polydispersity index (PDI) of RGD-Chi@tPA as a function of the molar ratio of RGD-Chi to tPA (RGD-Chi solution at pH 3.5 and 25 °C). Data are presented as the average ± standard deviation (n = 3).

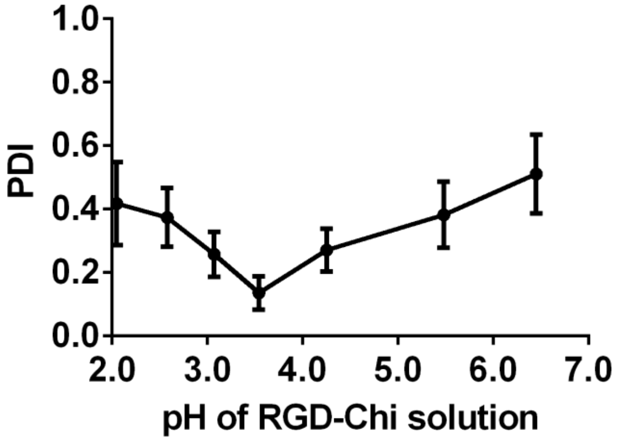

Figure S7. The PDI of RGD-Chi@tPA as a function of pH of RGD-Chi solution (1:1 molar ratio of RGD-Chi to tPA, 25 °C). Data are presented as the average ± standard deviation (n = 3).

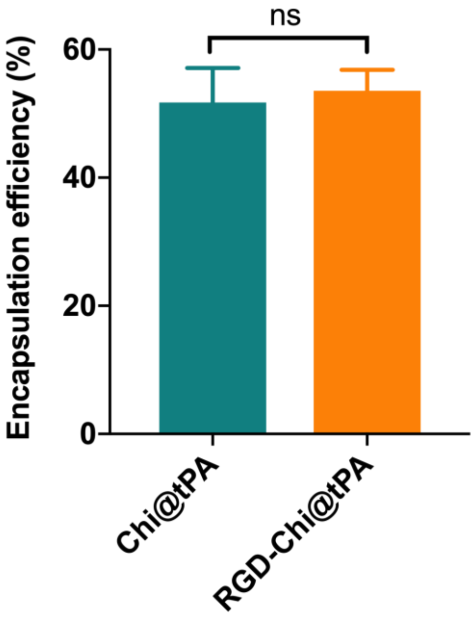

Figure S8. tPA encapsulation efficiency of Chi@tPA and RGD-Chi@tPA nanocoacervates. Statistical analysis was performed by the Student's *t*-test (ns: not significant).

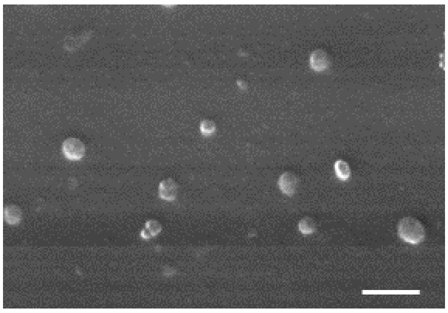

Figure S9. SEM micrograph of Chi@tPA nanocoacervates (Scale bar: 500 nm).

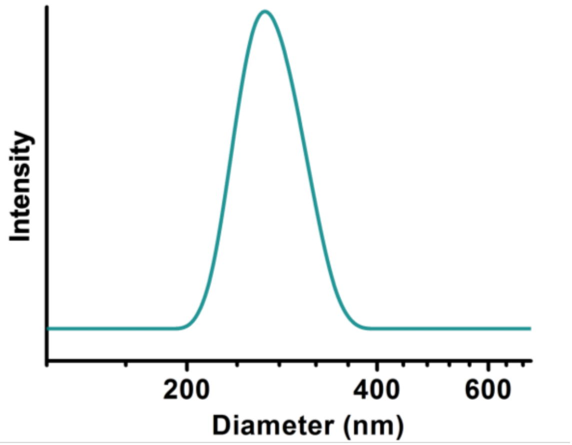

Figure S10. Typical intensity-weighted DLS plot of Chi@tPA in aqeuous solution (pH 7.4, 25 °C).

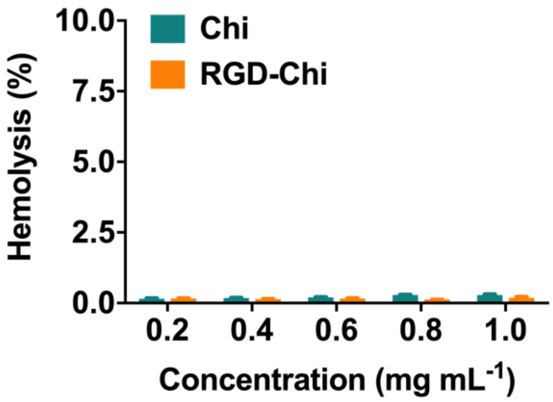

Figure S11. Hemolysis as a function of concentrations of Chi and RGD-Chi. Data are presented as the average ± standard deviation (n = 3).

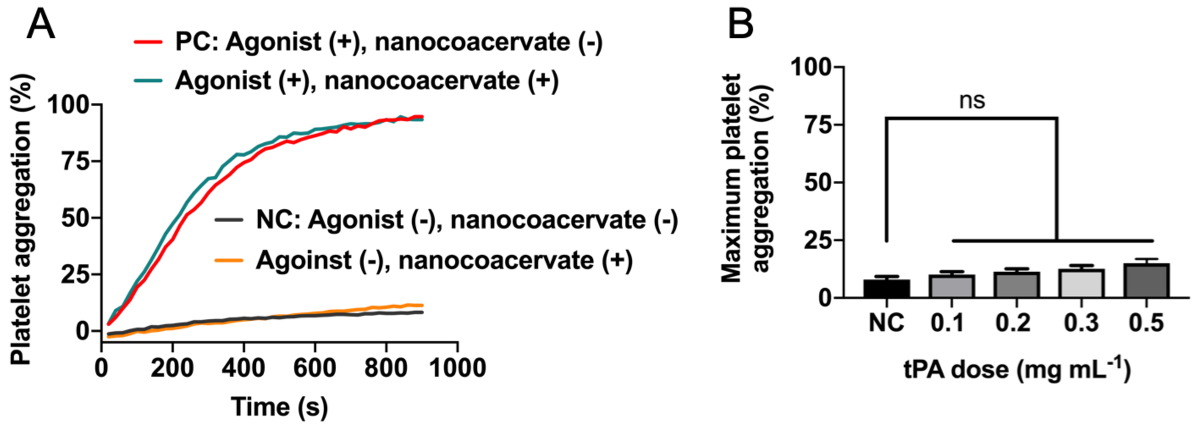

Figure S12. (A) Time-dependent platelet aggregation following pre-incubation with the RGD-Chi@tPA nanocoacervate (tPA dose of 0.2 mg mL^{-1}) at 37°C for 30 min with (blue curve) or without (yellow curve) stimulation by thrombin as an agonist at concentration of 1 U mL^{-1}. The platelets in the absence of RGD-Chi@tPA with or without simulation by the agonist were used as the positive control (PC, red curve) and negative control (NC, black curve), respectively. (B) Maximum platelet aggregation after incubation with RGD-Chi@tPA at different tPA doses for 15 min in the absence of agonist. The platelets in the absence of both RGD-Chi@tPA and agonist were used as the negative control (NC). Data are presented as the average ± standard deviation (n = 3). Statistical analysis was performed by the ANOVA test (ns: not significant).

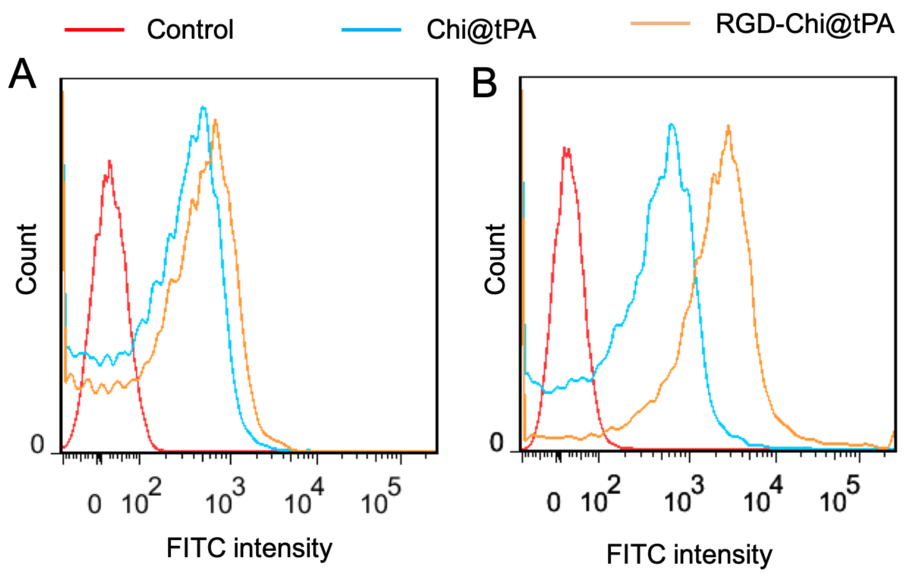

Figure S13. Flow cytometry histogram profiles of (A) resting platelets and (B) activated platelets incubated with the FITC-labeled Chi@tPA and RGD-Chi@tPA, respectively. The PBS buffer at pH 7.4 was used as a control.

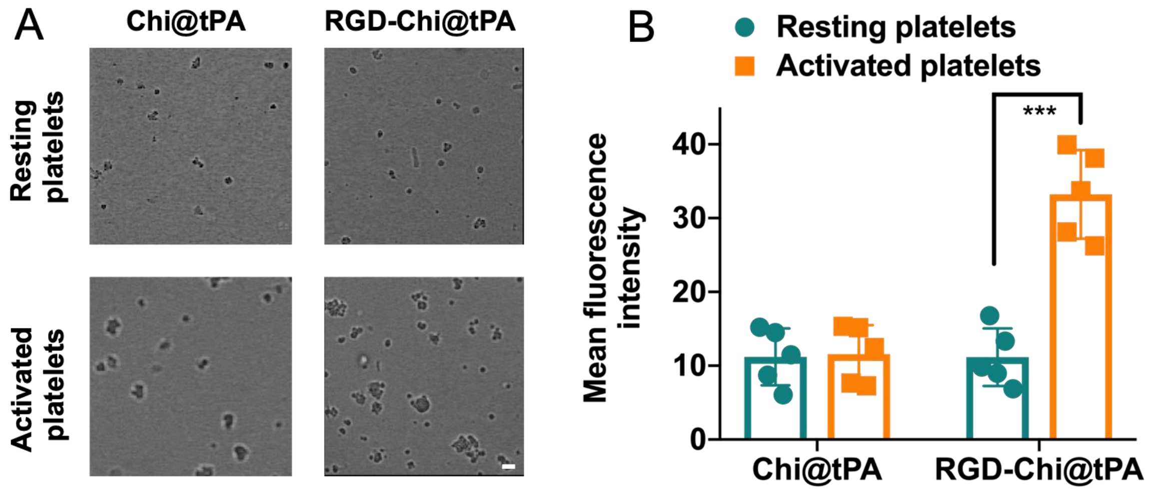

Figure S14. (A) Bright field images (scale bar: 5 μm) and (B) mean fluorescence intensity of FITC in the CLSM images (see **Figure 2B**) of resting and activated platelets after incubation with the FITC-labeled Chi@tPA and RGD-Chi@tPA, respectively, as analyzed by Fiji. Data are presented as the average ± standard deviation (n = 5). Statistical analysis was performed by the Student's *t*-test (***: $P < 0.001$).

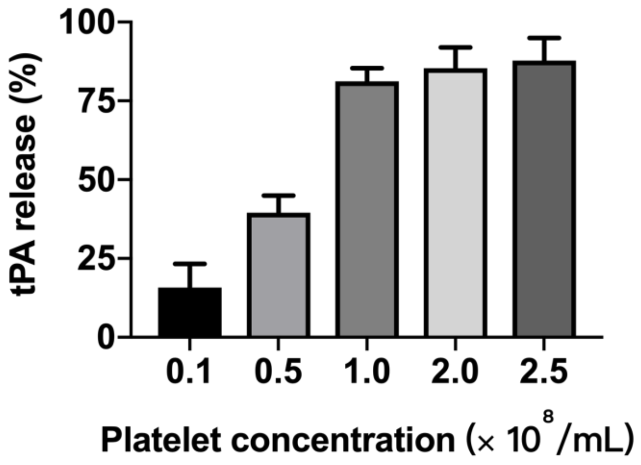

Figure S15. tPA release profiles of RGD-Chi@tPA after incubation with different concentrations of activated platelets for 2 h. Data are presented as the average ± standard deviation (n = 3).

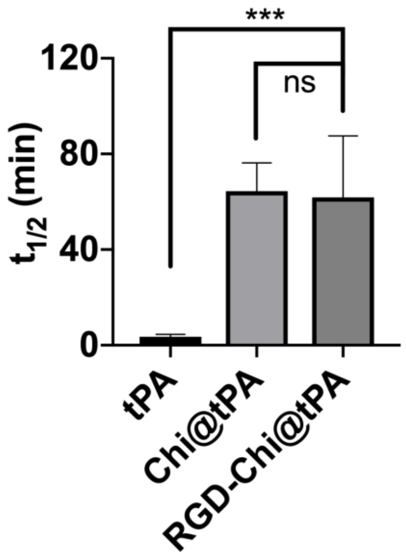

Figure S16. Half-lives ($t_{1/2}$) of free, tPA Chi@tPA and RGD-Chi@tPA in healthy SD rats. Data are presented as the average ± standard deviation (n = 3). Statistical analysis was performed by the Student's *t*-test and ANOVA test (***: $P < 0.001$ and ns: not significant).

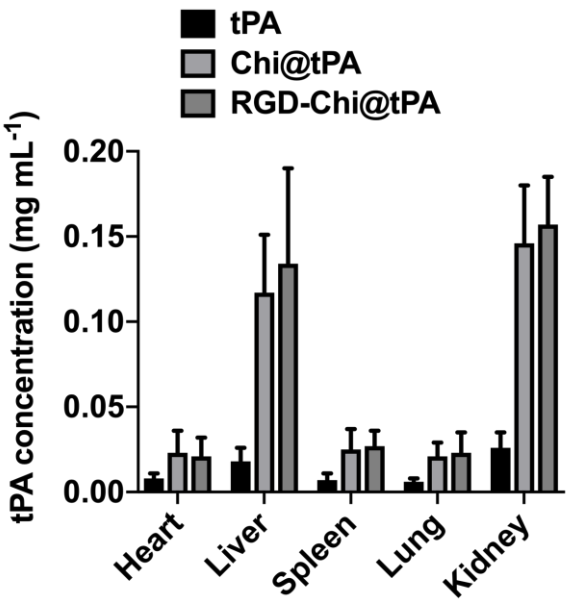

Figure S17. Tissue distribution of tPA in healthy SD rats after intravenous injection of the FITC-labeled free tPA, Chi@tPA and RGD-Chi@tPA for 4 h, respectively. Data are presented as the average ± standard deviation (n = 3).

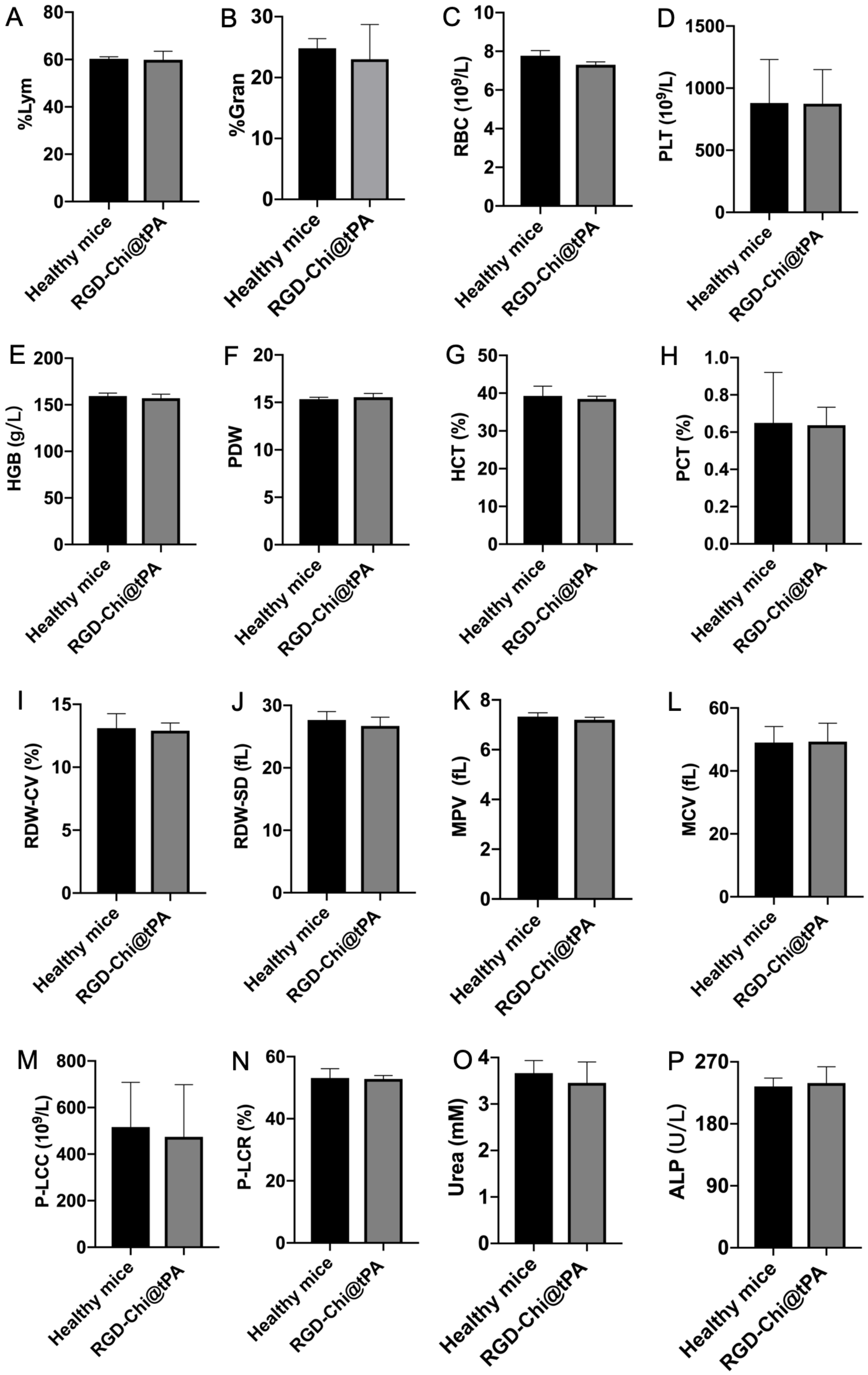

Figure S18. (A-N) Hematology indexes (lymphocytes percentage: %Lym, granulocytes percentage: %Gran, red blood cell: RBC, platelet: PLT, hemoglobin: HGB, platelet distribution width: PDW, hematocrit value: HCT, thrombocytocrit: PCT, coefficient of

variation of red blood cell volume distribution width: RDW-CV, standard deviation of red blood cell volume distribution width: RDW-SD, mean platelet volume: MPV, mean corpuscular volume: MCV, platelet large cell count: P-LCC, and platelet large cell ratio: P-LCR) and (O-P) biochemical indexes of Urea and alkaline phosphatase (ALP) in KM mice after 7 days of treatment with RGD-Chi@tPA (healthy KM mice as control). Data are presented as the average ± standard deviation (n = 3).

References

[1] S. Chen, R. Chen, *ACS Appl. Mater. Interfaces* **2016**, *8*, 22457.

[2] B. Vaidya, M. K. Nayak, D. Dash, G. P. Agrawal, S. P. Vyas, *Int. J. Pharm.* **2011**, *403*, 254.

[3] Y. Huang, B. Gu, I. I. Salles-Crawley, K. A. Taylor, L. Yu, J. Ren, X. Liu, M. Emerson, C. Longstaff, A. D. Hughes, S. A. Thom, X. Y. Xu, R. Chen, *Sci. Adv.* **2021**, *7*, eabf9033.

[4] Y. Huang, L. Yu, J. Ren, B. Gu, C. Longstaff, A. D. Hughes, S. A. Thom, X. Y. Xu, R. Chen, *J. Control. Release* **2019**, *300*, 1.

[5] S. Absar, K. Nahar, Y. M. Kwon, F. Ahsan, *Pharm. Res.* **2013**, *30*, 1663.

[6] T. Bonnard, L. S. Law, Z. Tennant, C. E. Hagemeyer, *Sci. Rep.* **2017**, *7*, 2346.

[7] C. Longstaff, M. Locke, *J. Thromb. Haemost.* **2019**, *17*, 195.
